# Splenic trauma: WSES classification and guidelines for adult and pediatric patients

**DOI:** 10.1186/s13017-017-0151-4

**Published:** 2017-08-18

**Authors:** Federico Coccolini, Giulia Montori, Fausto Catena, Yoram Kluger, Walter Biffl, Ernest E. Moore, Viktor Reva, Camilla Bing, Miklosh Bala, Paola Fugazzola, Hany Bahouth, Ingo Marzi, George Velmahos, Rao Ivatury, Kjetil Soreide, Tal Horer, Richard ten Broek, Bruno M. Pereira, Gustavo P. Fraga, Kenji Inaba, Joseph Kashuk, Neil Parry, Peter T. Masiakos, Konstantinos S. Mylonas, Andrew Kirkpatrick, Fikri Abu-Zidan, Carlos Augusto Gomes, Simone Vasilij Benatti, Noel Naidoo, Francesco Salvetti, Stefano Maccatrozzo, Vanni Agnoletti, Emiliano Gamberini, Leonardo Solaini, Antonio Costanzo, Andrea Celotti, Matteo Tomasoni, Vladimir Khokha, Catherine Arvieux, Lena Napolitano, Lauri Handolin, Michele Pisano, Stefano Magnone, David A. Spain, Marc de Moya, Kimberly A. Davis, Nicola De Angelis, Ari Leppaniemi, Paula Ferrada, Rifat Latifi, David Costa Navarro, Yashuiro Otomo, Raul Coimbra, Ronald V. Maier, Frederick Moore, Sandro Rizoli, Boris Sakakushev, Joseph M. Galante, Osvaldo Chiara, Stefania Cimbanassi, Alain Chichom Mefire, Dieter Weber, Marco Ceresoli, Andrew B. Peitzman, Liban Wehlie, Massimo Sartelli, Salomone Di Saverio, Luca Ansaloni

**Affiliations:** 1 General, Emergency and Trauma Surgery, Papa Giovanni XXIII Hospital, P.zza OMS 1, 24128 Bergamo, Italy; 2 Emergency and Trauma Surgery, Maggiore Hospital, Parma, Italy; 3 Division of General Surgery, Rambam Health Care Campus, Haifa, Israel; 4 Acute Care Surgery, The Queen’s Medical Center, Honolulu, HI USA; 5 Trauma Surgery, Denver Health Medical Center, Denver, CO USA; 6 General and Emergency Surgery, Sergei Kirov Military Academy, Saint Petersburg, Russia; 7 General and Emergency Surgery Department, Empoli Hospital, Empoli, Italy; 8 General and Emergency Surgery, Hadassah Medical Center, Jerusalem, Israel; 9 Klinik für Unfall-, Hand- und Wiederherstellungschirurgie Universitätsklinikum Goethe-Universität Frankfurt, Frankfurt, Germany; 10 Trauma, Emergency Surgery, and Surgical Critical Care, Massachusetts General Hospital, Boston, MA USA; 11 Virginia Commonwealth University, Richmond, VA USA; 12 Department of Gastrointestinal Surgery, Stavanger University Hospital, Stavanger, Norway; 13 Department of Cardiothoracic and Vascular Surgery, Örebro University Hospital and Örebro University, Orebro, Sweden; 14 Department of Surgery, Radboud University Nijmegen Medical Center, Nijmegen, Netherlands; 15 Trauma/Acute Care Surgery and Surgical Critical Care, University of Campinas, Campinas, Brazil; 16 Division of Trauma and Critical Care, LAC+USC Medical Center, Los Angeles, CA USA; 17 Department of Surgery, Assia Medical Group, Tel Aviv University Sackler School of Medicine, Tel Aviv, Israel; 18 General and Trauma Surgery Department, London Health Sciences Centre, Victoria Hospital, London, ON Canada; 19 Pediatric Trauma Service, Massachusetts General Hospital, Boston, MA USA; 20 General, Acute Care, Abdominal Wall Reconstruction, and Trauma Surgery, Foothills Medical Centre, Calgary, AB Canada; 21 Department of Surgery, College of Medicine and Health Sciences, UAE University, Al-Ain, United Arab Emirates; 22 Universidade Federal de Juiz de Fora, Juiz de Fora, Brazil; 23 Infectivolgy Department, Papa Giovanni XXIII Hospital, Bergamo, Italy; 24 Department of Surgery, University of KwaZulu-Natal, Durban, South Africa; 25 Anesthesia Department, Bufalini Hospital, Cesena, Italy; 26 General Surgery Department, Mozir City Hospital, Mozir, Belarus; 27 Clin. Univ. de Chirurgie Digestive et de l’Urgence, CHUGA-CHU Grenoble Alpes UGA-Université Grenoble Alpes, Grenoble, France; 28 Trauma and Surgical Critical Care, University of Michigan Health System, East Medical Center Drive, Ann Arbor, MI USA; 29 Trauma Unit, Helsinki University Hospital, Helsinki, Finland; 30 Department of Surgery, Stanford University, Stanford, CA USA; 31 General Surgery, Trauma, and Surgical Critical Care, Yale-New Haven Hospital, New Haven, CT USA; 32 Hopital Heri Mondor, Paris, France; 33 General Surgery Department, Mehilati Hospital, Helsinki, Finland; 34 General Surgery Department, Westchester Medical Center, Westchester, NY USA; 35 Colorectal Surgery Unit, Trauma Care Committee, Alicante General University Hospital, Alicante, Spain; 36 Trauma and Acute Critical Care Center, Tokyo Medical and Dental University, Tokyo, Japan; 37 Department of Surgery, UC San Diego Health System, San Diego, USA; 38 Department of Surgery, University of Washington, Seattle, WA USA; 39 Department of Surgery, Gainesville, FL USA; 40 Trauma and Acute Care Service, St Michael’s Hospital, Toronto, ON Canada; 41 General Surgery Department, Medical University, University Hospital St George, Plovdiv, Bulgaria; 42 Division of Trauma and Acute Care Surgery, University of California, Davis Medical Center, Davis, CA USA; 43 Trauma Team, Ospedale Niguarda, Milan, Italy; 44 Department of Surgery and Obstetric and Gynecology, University of Buea, Buea, Cameroon; 45 Department of General Surgery, Royal Perth Hospital, Perth, Australia; 46 Surgery Department, University of Pittsburgh, Pittsburgh, Pensylvania USA; 47 General Surgery Department, Ayaan Hospital, Mogadisho, Somalia; 48 General and Emergency Surgery, Macerata Hospital, Macerata, Italy; 49 General, Emergency and Trauma Surgery Department, Maggiore Hospital, Bologna, Italy; 50 Department of Surgery, Örebro University Hospital and Örebro University, Obreo, Sweden

**Keywords:** Spleen, Trauma, Adult, Pediatric, Classification, Guidelines, Embolization, Surgery, Non-operative, Conservative

## Abstract

Spleen injuries are among the most frequent trauma-related injuries. At present, they are classified according to the anatomy of the injury. The optimal treatment strategy, however, should keep into consideration the hemodynamic status, the anatomic derangement, and the associated injuries. The management of splenic trauma patients aims to restore the homeostasis and the normal physiopathology especially considering the modern tools for bleeding management. Thus, the management of splenic trauma should be ultimately multidisciplinary and based on the physiology of the patient, the anatomy of the injury, and the associated lesions. Lastly, as the management of adults and children must be different, children should always be treated in dedicated pediatric trauma centers. In fact, the vast majority of pediatric patients with blunt splenic trauma can be managed non-operatively. This paper presents the World Society of Emergency Surgery (WSES) classification of splenic trauma and the management guidelines.

## Background

The management of splenic trauma has changed considerably in the last few decades especially in favor of non-operative management (NOM). NOM ranges from observation and monitoring alone to angiography/angioembolization (AG/AE) with the aim to preserve the spleen and its function, especially in children. These considerations were carried out considering the immunological function of the spleen and the high risk of immunological impairment in splenectomized patients. In contrast with liver traumatic injuries, splenic injuries can be fatal not only at the admission of the patient to the Emergency Department (ED), but also due to delayed subcapsular hematoma rupture or pseudoaneurism (PSA) rupture. Lastly, overwhelming post-splenectomy infections (OPSI) are a late cause of complications due to the lack of the immunological function of the spleen. For these reasons, standardized guidelines in the management of splenic trauma are necessary.

The existing classification of splenic trauma considered the anatomical lesions (Table 1). However, patients’ conditions may lead to an emergent transfer to the operating room (OR) without the opportunity to define the grade of the splenic lesions before the surgical exploration. This confirms the primary importance of the patient’s overall clinical condition in these settings. In addition, the modern tools in bleeding management have helped in adopting a conservative approach also in severe lesions. Trauma management must be multidisciplinary and requires an assessment of both the anatomical injury and its physiologic effects. The present guidelines and classification reconsider splenic lesions in the light of the physiopathologic status of the patient associated with the anatomic grade of injury and the other associated lesions.

**Table 1:** AAST Spleen Trauma Classification

Grade	Injury description	
I	Hematoma	Subcapsular, < 10% surface area
	Laceration	Capsular tear, < 1 cm parenchymal depth
II	Hematoma	Subcapsular, 10–50% surface area
		Intraparenchymal, < 5 cm diameter
	Laceration	1–3 cm parenchymal depth not involving a perenchymal vessel
III	Hematoma	Subcapsular, > 50% surface area or expanding
		Ruptured subcapsular or parenchymal hematoma
		Intraparenchymal hematoma > 5 cm
	Laceration	> 3 cm parenchymal depth or involving trabecular vessels
IV	Laceration	Laceration of segmental or hilar vessels producing major devascularization (> 25% of spleen)
V	Laceration	Completely shatters spleen
	Vascular	Hilar vascular injury which devascularized spleen

### Notes on the use of the guidelines

The guidelines are evidence-based, with the grade of recommendation also based on the evidence. The guidelines present the diagnostic and therapeutic methods for optimal management of spleen trauma. The practice guidelines promulgated in this work do not represent a standard of practice. They are suggested plans of care, based on best available evidence and the consensus of experts, but they do not exclude other approaches as being within the standard of practice. For example, they should not be used to compel adherence to a given method of medical management, which method should be finally determined after taking account of the conditions at the relevant medical institution (staff levels, experience, equipment, etc.) and the characteristics of the individual patient. However, responsibility for the results of treatment rests with those who are directly engaged therein, and not with the consensus group.

## Methods

**Fig. 1:**
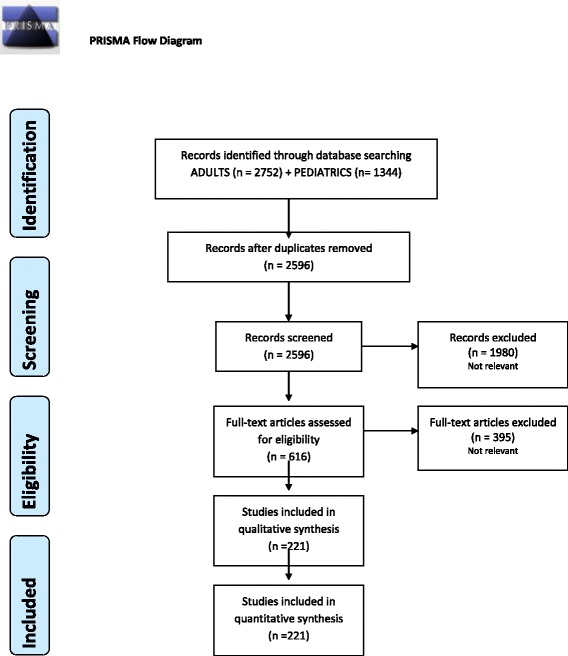
PRISMA flow chart

A computerized search was done by the bibliographer in different databanks (MEDLINE, Scopus, EMBASE) citations were included for the period between January 1980 and May 2016 using the primary search strategy: spleen, injuries, trauma, resuscitation, adult, pediatric, hemodynamic instability/stability, angioembolization, management, infection, follow-up, vaccination, and thrombo-prophylaxis combined with AND/OR. No search restrictions were imposed. The dates were selected to allow comprehensive published abstracts of clinical trials, consensus conference, comparative studies, congresses, guidelines, government publication, multicenter studies, systematic reviews, meta-analysis, large case series, original articles, and randomized controlled trials. Case reports and small cases series were excluded. Narrative review articles were also analyzed to determine other possible studies. Literature selection is reported in the flow chart (Fig. 1). The Level of evidence (LE) was evaluated using the GRADE system [1] (Table 2).

**Table 2:** GRADE system to evaluate the level of evidence and recommendation

Grade of recommendation	Clarity of risk/benefit	Quality of supporting evidence	Implications
1A			
Strong recommendation, high-quality evidence	Benefits clearly outweigh risk and burdens, or vice versa	RCTs without important limitations or overwhelming evidence from observational studies	Strong recommendation, applies to most patients in most circumstances without reservation
1B			
Strong recommendation, moderate-quality evidence	Benefits clearly outweigh risk and burdens, or vice versa	RCTs with important limitations (inconsistent results, methodological flaws, indirect analyses or imprecise conclusions) or exceptionally strong evidence from observational studies	Strong recommendation, applies to most patients in most circumstances without reservation
1C			
Strong recommendation, low-quality or very low-quality evidence	Benefits clearly outweigh risk and burdens, or vice versa	Observational studies or case series	Strong recommendation but subject to change when higher quality evidence becomes available
2A			
Weak recommendation, high-quality evidence	Benefits closely balanced with risks and burden	RCTs without important limitations or overwhelming evidence from observational studies	Weak recommendation, best action may differ depending on the patient, treatment circumstances, or social values
2B			
Weak recommendation, moderate-quality evidence	Benefits closely balanced with risks and burden	RCTs with important limitations (inconsistent results, methodological flaws, indirect or imprecise) or exceptionally strong evidence from observational studies	Weak recommendation, best action may differ depending on the patient, treatment circumstances, or social values
2C			
Weak recommendation, low-quality or very low-quality evidence	Uncertainty in the estimates of benefits, risks, and burden; benefits, risk, and burden may be closely balanced	Observational studies or case series	Very weak recommendation; alternative treatments may be equally reasonable and merit consideration

A group of experts in the field coordinated by a central coordinator was contacted to express their evidence-based opinion on several issues about the pediatric (< 15 years old) and adult splenic trauma. Splenic trauma were divided and assessed as type of injury (blunt and penetrating injury) and management (conservative and operative management). Through the Delphi process, the different issues were discussed in subsequent rounds. The central coordinator assembled the different answers derived from each round. Each version was then revised and improved. The definitive version was discussed during the WSES World Congress in May 2017 in Campinas, Brazil. The final version about which the agreement was reached resulted in present paper.

### WSES classification

The WSES position paper suggested to group splenic injury into minor, moderate, and severe. This classification has not previously been clearly defined by the literature. Frequently low-grade AAST lesions (i.e., grades I–III) are considered as minor or moderate and treated with NOM. However, hemodynamically stable patients with high-grade lesions could be successfully treated non-operatively, especially exploiting the more advanced tools for bleeding management. On the other hand, “minor” lesions associated with hemodynamic instability often must be treated with OM. This demonstrates that the classification of spleen injuries into minor and major must consider both the anatomic AAST-OIS classification and the hemodynamic status.

The WSES classification divides spleen injuries into three classes:

- Minor (WSES class I)
- Moderate (WSES classes II and III)
- Severe (WSES class IV)

The classification considers the AAST-OIS classification and the hemodynamic status and is the same for adult and pediatric patients. Table 3 explains the classification with the different key points of treatment differentiated within adult and pediatric patients; Table 4 resumes the guidelines statements.

**Table 3:** WSES Spleen Trauma Classification for adult and pediatric patients

	WSES class	Mechanism of injury	AAST	Hemodynamic status^a, b^	CT scan	First-line treatment in adults	First-line treatment in pediatric
Minor	WSES I	Blunt/penetrating	I–II	Stable	Yes + local exploration in SW^d^	NOM^c^ + serial clinical/laboratory/radiological evaluation Consider angiography/angioembolization	NOM^c^ + serial clinical/laboratory/radiological evaluation Consider angiography/angioembolization
Moderate	WSES II	Blunt/penetrating	III	Stable			
	WSES III	Blunt/penetrating	IV–V	Stable		NOM^c^ All angiography/angioembolization + serial clinical/laboratory/radiological evaluation	
Severe	WSES IV	Blunt/penetrating	I–V	Unstable	No	OM	OM

*SW* stab wound, *GSW* gunshot wound

^**a**^ *Hemodynamic instability in adults* is considered the condition in which the patient has an admission systolic blood pressure < 90 mmHg with evidence of skin vasoconstriction (cool, clammy, decreased capillary refill), altered level of consciousness and/or shortness of breath, or > 90 mmHg but requiring bolus infusions/transfusions and/or vasopressor drugs and/or admission base excess (BE) > − 5 mmol/l and/or shock index > 1 and/or transfusion requirement of at least 4–6 units of packed red blood cells within the first 24 h; moreover, transient responder patients (those showing an initial response to adequate fluid resuscitation, and then signs of ongoing loss and perfusion deficits) and more in general those responding to therapy but not amenable of sufficient stabilization to be undergone to interventional radiology treatments

^b^ *Hemodynamic stability in pediatric patients* is considered systolic blood pressure of 90 mmHg plus twice the child’s age in years (the lower limit is inferior to 70 mmHg plus twice the child’s age in years, or inferior to 50 mmHg in some studies). Stabilized or acceptable hemodynamic status is considered in children with a positive response to fluid resuscitation: 3 boluses of 20 mL/kg of crystalloid replacement should be administered before blood replacement; positive response can be indicated by the heart rate reduction, the sensorium clearing, the return of peripheral pulses and normal skin color, an increase in blood pressure and urinary output, and an increase in warmth of extremity. Clinical judgment is fundamental in evaluating children

^c^NOM should only be attempted in centers capable of a precise diagnosis of the severity of spleen injuries and capable of intensive management (close clinical observation and hemodynamic monitoring in a high dependency/intensive care environment, including serial clinical examination and laboratory assay, with immediate access to diagnostics, interventional radiology, and surgery and immediately available access to blood and blood products or alternatively in the presence of a rapid centralization system in those patients amenable to be transferred

^d^Wound exploration near the inferior costal margin should be avoided if not strictly necessary because of the high risk to damage the intercostal vessels

**Table 4:** Statement summary

	Adults	Pediatrics
Diagnostic procedures	-The choice of diagnostic technique at admission must be based on the hemodynamic status of the patient (GoR 1A). -E-FAST is effective and rapid to detect free fluid (GoR 1A). -CT scan with intravenous contrast is the gold standard in hemodynamically stable or stabilized trauma patients (GoR 1A). -Doppler US and contrast-enhanced US are useful to evaluate splenic vascularization and in follow-up (GoR 1B). -Injury grade on CT scan, extent of free fluid, and the presence of PSA do not predict NOM failure or the need of OM (GoR 1B).	-The role of E-FAST in the diagnosis of pediatric spleen injury is still unclear (GoR 1A). -A positive E-FAST examination in children should be followed by an urgent CT in stable patients (GoR 1B). -Complete abdominal US may avoid the use of CT in stable patients (GoR 1B). -Contrast-enhanced CT scan is the gold standard in pediatric splenic trauma (GoR 1A). -Doppler US and contrast-enhanced US are useful to evaluate splenic vascularization (GoR 1B). -CT scan is suggested in children at risk for head and thoracic injuries, need for surgery, recurrent bleeding, and if other abdominal injuries are suspected (GoR 1A). -Injury grade on CT scan, free fluid amount, contrast blush, and the presence of pseudo-aneurysm do not predict NOM failure or the need for OM (GoR 1B).
Non-operative management • General indications		-NOM is recommended as first-line treatment for hemodynamically stable pediatric patients with blunt splenic trauma (GoR 2A). -Patients with moderate-severe blunt and all penetrating splenic injuries should be considered for transfer to dedicated pediatric trauma centers after hemodynamic stabilization (GoR2A). -NOM of spleen injuries in children should be considered only in an environment that provides capability for patient continuous monitoring, angiography, and trained surgeons, an immediately available OR and immediate access to blood and blood products or alternatively in the presence of a rapid centralization system in those patients amenable to be transferred (GoR 2A). -NOM should be attempted even in the setting of concomitant head trauma; unless the patient is unstable, this might be due to intra-abdominal bleeding (GoR 2B).
• Blunt/penetrating trauma	-Patients with hemodynamic stability and absence of other abdominal organ injuries requiring surgery should undergo an initial attempt of NOM irrespective of injury grade (GoR 2A). -NOM of moderate or severe spleen injuries should be considered only in an environment that provides capability for patient intensive monitoring, AG/AE, an immediately available OR and immediate access to blood and blood product or alternatively in the presence of a rapid centralization system and only in patients with stable or stabilized hemodynamic and absence of other internal injuries requiring surgery (GoR 2A). -NOM in splenic injuries is contraindicated in the setting of unresponsive hemodynamic instability or other indicates for laparotomy (peritonitis, hollow organ injuries, bowel evisceration, impalement) (GoR 1A). -In patients being considered for NOM, CT scan with intravenous contrast should be performed to define the anatomic spleen injury and identify associated injuries (GoR 2A). -AG/AE may be considered the first-line intervention in patients with hemodynamic stability and arterial blush on CT scan irrespective from injury grade (GoR 2B). -Strong evidence exists that age above 55 years old, high ISS, and moderate to severe splenic injuries are prognostic factors for NOM failure. These patients require more intensive monitoring and higher index of suspicion (GoR 2B). -Age above 55 years old alone, large hemoperitoneum alone, hypotension before resuscitation, GCS < 12 and low-hematocrit level at the admission, associated abdominal injuries, blush at CT scan, anticoagulation drugs, HIV disease, drug addiction, cirrhosis, and need for blood transfusions should be taken into account, but they are not absolute contraindications for NOM (GoR 2B). -In WSES class II–III spleen injuries with associated severe traumatic brain injury, NOM could be considered only if rescue therapy (OR and/or AG/AE) is rapidly available; otherwise, splenectomy should be performed (GoR 1C).	Blunt trauma -Blunt splenic injuries with hemodynamic stability and absence of other internal injuries requiring surgery, should undergo an initial attempt of NOM irrespective of injury grade (GoR 2A). -In hemodynamically stable children with isolated splenic injury splenectomy should be avoided (GoR 1A). -NOM is contraindicated in presence of peritonitis, bowel evisceration, impalement or other indications to laparotomy (GoR 2A). -The presence of contrast blush at CT scan is not an absolute indication for splenectomy or AG/AE in children (GoR 2B). Intensive care unit admission in isolated splenic injury may be required only for moderate and severe lesions (GoR 2B).
		Penetrating trauma -No sufficient data validating NOM for penetrating spleen injury in children exist.
The role of angiography/angioembolization (AG/AE)	-AG/AE may be performed in hemodynamically stable and rapid responder patients with moderate and severe lesions and in those with vascular injuries at CT scan (contrast blush, pseudo-aneurysms and arterio-venous fistula) (GoR 2A). -In patients with bleeding vascular injuries and in those with intraperitoneal blush, AG/AE should be performed as part of NOM only in centers where AG/AE is rapidly available. In other centers and in case of rapid hemodynamic deterioration, OM should be considered (GoR 2B). -In case of absence of blush during angiography, if blush was previously seen at CT scan, proximal angioembolization could be considered (GoR 2C). –AG/AE should be considered in all hemodynamically stable patients with WSES grade III lesions, regardless with the presence of CT blush (GoR 1B). –AG/AE could be considered in patients undergone to NOM, hemodynamically stable with sings of persistent hemorrhage regardless with the presence of CT blush once excluded extra-splenic source of bleeding (GoR 1C). –Hemodynamically stable patients with WSES grade II lesions without blush should not underwent routine AG/AE but may be considered for prophylactic proximal embolization in presence of risk factors for NOM failure (GoR 2B). –In the presence of a single vascular abnormality (contrast blush, pseudo-aneurysms, and artero-venous fistula) in minor and moderate injuries, the currently available literature is inconclusive regarding whether proximal or distal embolization should be used. In the presence of multiple splenic vascular abnormalities or in the presence of a severe lesion, proximal or combined AG/AE should be used, after confirming the presence of a permissive pancreatic vascular anatomy (GoR 1C). –In performing, AG/AE coils should be preferred to temporary agents (GoR 1C).	-The vast majority of pediatric patients do not require AG/AE for CT blush or moderate to severe injuries (GoR 1C). -AG/AE may be considered in patients undergone to NOM, hemodynamically stable with sings of persistent hemorrhage not amenable of NOM, regardless with the presence of CT blush once excluded extra-splenic source of bleeding (GoR 1C). -AG/AE may be considered for the treatment of post-traumatic splenic pseudo-aneurysms prior to patient discharge (GoR 2C). -Patients with more than 15 years old should be managed according to adults AG/AE-protocols (GoR 1C).
Operative management (OM)	-OM should be performed in patients with hemodynamic instability and/or with associated lesions like peritonitis or bowel evisceration or impalement requiring surgical exploration (GoR 2A). -OM should be performed in moderate and severe lesions even in stable patients in centers where intensive monitoring cannot be performed and/or when AG/AE is not rapidly available (GoR 2A). -Splenectomy should be performed when NOM with AG/AE failed, and patient remains hemodynamically unstable or shows a significant drop in hematocrit levels or continuous transfusion are required (GoR 2A). –During OM, salvage of at least a part of the spleen is debated and could not be suggested (GoR 2B). –Laparoscopic splenectomy in early trauma scenario in bleeding patients could not be recommended (GoR 2A).	-Patients should undergo to OM in case of hemodynamic instability, failure of conservative treatments, severe coexisting injuries necessitating intervention and peritonitis, bowel evisceration, impalement (GoR 2A). -Splenic preservation (at least partial) should be attempted whenever possible (GoR 2B).
Short- and long-term follow-up	–Clinical and laboratory observation associated to bed rest in moderate and severe lesions is the cornerstone in the first 48–72 h follow-up (GoR 1C). –CT scan repetition during the admission should be considered in patients with moderate and severe lesions or in decreasing hematocrit, in presence of vascular anomalies or underlying splenic pathology or coagulopathy, and in neurologically impaired patients (GoR 2A). –In the presence of underlying splenic pathology or coagulopathy and in neurologically impaired patients CT follow-up is to be considered after the discharge (GoR 2B). –Activity restriction may be suggested for 4–6 weeks in minor injuries and up to 2–4 months in moderate and severe injuries (GoR 2C).	–In hemodynamic stable children without drop in hemoglobin levels for 24 h, bed rest should be suggested (GoR 2B). –The risk of pseudo-aneurysm after splenic trauma is low, and in most of cases, it resolves spontaneously (GoR 2B). –Angioembolization should be taken into consideration when a pesudoaneurysm is found (GoR 2B). –US (DUS, CEUS) follow-up seems reasonable to minimize the risk of life-threatening hemorrhage and associated complications in children (GoR 1B). –After NOM in moderate and severe injuries, the reprise of normal activity could be considered safe after at least 6 weeks (GoR 2B).
Thrombo-prophylaxis	–Mechanical prophylaxis is safe and should be considered in all patients without absolute contraindication to its use (GoR 2A). – Spleen trauma without ongoing bleeding is not an absolute contraindication to LMWH-based prophylactic anticoagulation (GoR 2A) –LMWH-based prophylactic anticoagulation should be started as soon as possible from trauma and may be safe in selected patients with blunt splenic injury undergone to NOM (GoR 2B). –In patient with oral anticoagulants the risk-benefit balance of reversal should be individualized (GoR 1C).	
Infections prophylaxis in asplenic and hyposplenic adult and pediatric patients	–Patients should receive immunization against the encapsulated bacteria (*S. pneumoniae*, *H. influenzae*, and *N. meningitidis*) (GoR 1A). –Vaccination programs should be started no sooner than 14 days after splenectomy or spleen total vascular exclusion (GoR 2C). –In patients discharged before 15 days after splenectomy or angioembolization, where the risk to miss vaccination is deemed high, the best choice is to vaccinate before discharge (GoR 1B). –Immunization against seasonal flu is recommended for patients over 6 months of age (GoR 1C). –Malaria prophylaxis is strongly recommended for travelers (GoR 2C). –Antibiotic therapy should be strongly considered in the event of any sudden onset of unexplained fever, malaise, chills or other constitutional symptoms, especially when medical review is not readily accessible (GoR 2A). –Primary care providers should be aware of the splenectomy/angioembolization (GoR 2C).	

*Minor spleen injuries:*

- *WSES class I* includes hemodynamically stable AAST-OIS grade I–II blunt and penetrating lesions.

*Moderate spleen injuries:*

- *WSES class II* includes hemodynamically stable AAST-OIS grade III blunt and penetrating lesions.
- *WSES class III* includes hemodynamically stable AAST-OIS grade IV–V blunt and penetrating lesions.

*Severe spleen injuries:*

- *WSES class IV* includes hemodynamically unstable AAST-OIS grade I–V blunt and penetrating lesions.

**Fig. 2:**
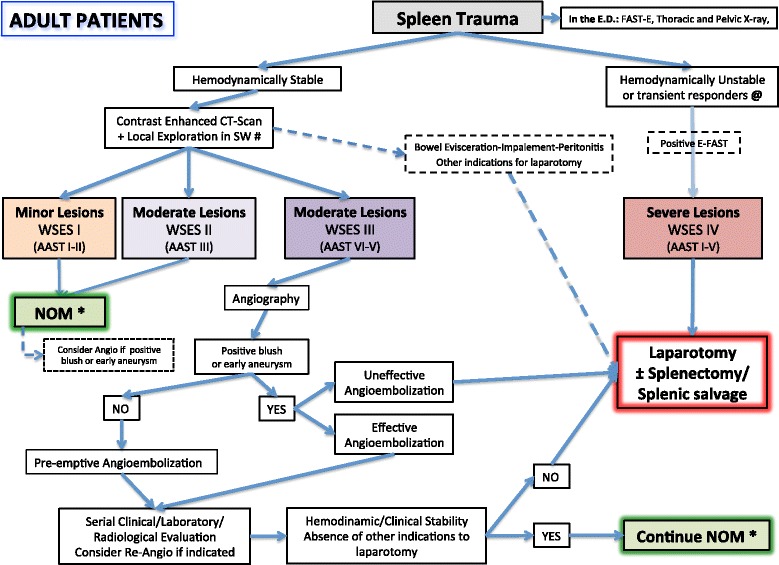
Spleen Trauma Management Algorithm for Adult Patients. (*SW* stab wound, *GSW* gunshot wound. *NOM should only be attempted in centers capable of a precise diagnosis of the severity of spleen injuries and capable of intensive management (close clinical observation and hemodynamic monitoring in a high dependency/intensive care environment, including serial clinical examination and laboratory assay, with immediate access to diagnostics, interventional radiology, and surgery and immediately available access to blood and blood products or alternatively in the presence of a rapid centralization system in those patients amenable to be transferred; **@** *Hemodynamic instability* is considered the condition in which the patient has an admission systolic blood pressure < 90 mmHg with evidence of skin vasoconstriction (cool, clammy, decreased capillary refill), altered level of consciousness and/or shortness of breath, or > 90 mmHg but requiring bolus infusions/transfusions and/or vasopressor drugs and/or admission base excess (BE) > − 5 mmol/l and/or shock index > 1 and/or transfusion requirement of at least 4–6 units of packed red blood cells within the first 24 h; moreover, transient responder patients (those showing an initial response to adequate fluid resuscitation, and then signs of ongoing loss and perfusion deficits) and more in general those responding to therapy but not amenable of sufficient stabilization to be undergone to interventional radiology treatments. ***#*** Wound exploration near the inferior costal margin should be avoided if not strictly necessary because of the high risk to damage the intercostal vessels)

**Fig. 3:**
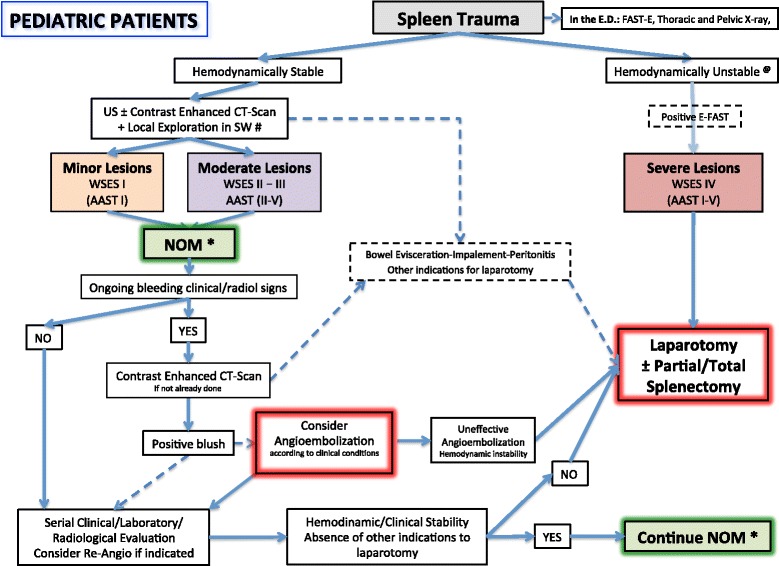
Spleen Trauma Management Algorithm for Pediatrics Patients. (*SW* stab wound, *GSW* gunshot wound; *NOM should only be attempted in centers capable of a precise diagnosis of the severity of spleen injuries and capable of intensive management (close clinical observation and hemodynamic monitoring in a high dependency/intensive care environment, including serial clinical examination and laboratory assay, with immediate access to diagnostics, interventional radiology, and surgery and immediately available access to blood and blood products or alternatively in presence of a rapid centralization system in those patients amenable to be transferred; **@** *Hemodynamic stability* is considered systolic blood pressure of 90 mmHg plus twice the child’s age in years (the lower limit is inferior to 70 mmHg plus twice the child’s age in years, or inferior to 50 mmHg in some studies). Stabilized or acceptable hemodynamic status is considered in children with a positive response to fluids resuscitation: 3 boluses of 20 mL/kg of crystalloid replacement should be administered before blood replacement; positive response can be indicated by the heart rate reduction, the sensorium clearing, the return of peripheral pulses and normal skin color, an increase in blood pressure and urinary output, and an increase in warmth of extremity. Clinical judgment is fundamental in evaluating children. ***#*** Wound exploration near the inferior costal margin should be avoided if not strictly necessary because of the high risk to damage the intercostal vessels)

Based on the present classification, WSES suggests two management algorithms for both adult and pediatric patients explained in Figs. 2 and 3.

## Adult patients

### Physiopathology of injuries

Some mechanisms of injuries are similar between children and adults like motor vehicle crashes and pedestrian accidents, while others like motorcycle accidents, sport injuries, gunshot or stab-related injuries, and assaults are more frequent in adults [2].

A few authors consider a normal hemodynamic status in adults when the patient does not require fluids or blood to maintain blood pressure, without signs of hypoperfusion; hemodynamic stability in adults as a counterpart is the condition in which the patient achieve a constant or an amelioration of blood pressure after fluids with a blood pressure > 90 mmHg and heart rate < 100 bpm; hemodynamic instability in adults is the condition in which the patient has an admission systolic blood pressure < 90 mmHg, or > 90 mmHg but requiring bolus infusions/transfusions and/or vasopressor drugs and/or admission base excess (BE) > −5 mmol/l and/or shock index > 1 [3, 4] and/or transfusion requirement of at least 4–6 units of packed red blood cells within the first 24 h [5]. The 9th edition of the Advanced Trauma Life Support (ATLS) definition considers as “unstable” the patient with the following: blood pressure < 90 mmHg and heart rate > 120 bpm, with evidence of skin vasoconstriction (cool, clammy, decreased capillary refill), altered level of consciousness and/or shortness of breath [5]. Moreover, transient responder patients (those showing an initial response to adequate fluid resuscitation and then signs of ongoing loss and perfusion deficits) and, more in general, those responding to therapy but not amenable of sufficient stabilization to be undergone to interventional radiology treatments, are to be considered as unstable patients. In the management of severe bleeding, the early evaluation and correction of the trauma-induced coagulopathy remains a main cornerstone. Physiologic impairment is frequently associated with aggressive resuscitation and the activation and deactivation of several procoagulant and anticoagulant factors contributes to the insurgence of trauma-induced coagulopathy. The application of massive transfusion protocols (MTP) is of paramount importance. The advanced tailored evaluation of the patient’s coagulative asset is clearly demonstrated as fundamental in driving the administration of blood products, coagulation factors, and drugs [6–9].

Diagnostic procedures:

- *The choice of diagnostic technique at admission must be based on the hemodynamic status of the patient (GoR 1A).*
- *E-FAST is effective and rapid to detect free fluid (GoR 1A).*
- *CT scan with intravenous contrast is the gold standard in hemodynamically stable or stabilized trauma patients (GoR 1A).*
- *Doppler US and contrast-enhanced US are useful to evaluate splenic vascularization and in follow-up (GoR 1B).*
- *Injury grade on CT scan, extent of free fluid, and the presence of PSA do not predict NOM failure or the need of OM (GoR 1B).*

Extended focused assessment sonography for trauma (E-FAST) and ultrasonography (US) have replaced diagnostic peritoneal lavage (DPL) management of abdominal trauma in present days [5, 10, 11]. Studies have shown a sensitivity up to 91% and a specificity up to 96% also for a small fluid amount [12, 13].

Nevertheless, 42% of false-negative have been reported [10]. This might be due to the 20% of cases in which no significant extravasation of blood is present in splenic trauma or in injuries near the diaphragm [10, 12, 13].

*Contrast-enhanced US (CEUS)* increases the visualization of a variety of splenic injuries and complications [12].

*Doppler US (DUS)* has been reported as safe and effective in evaluating PSA or blush previously found at CT scan [14].

Contrast tomography (CT) scan is considered the gold standard in trauma with a sensitivity and specificity for splenic injuries near to 96–100% [10, 15, 16]. However, Carr et al. [10] reported that CT scan can underestimate splenic injuries at ilum. CT must be rapidly available and must be performed only in hemodynamically stable patients or in those responding to fluid resuscitation [17, 18]. However, in some centers, there is the possibility to perform a fast-track CT scan that seems to permit to expand the criteria for performing CT scan in trauma patients. Delayed-phase CT helps in differentiating patients with active bleeding from those with contained vascular injuries [19]. This is important to reduce the risk of discrepancy between CT scan images and angio images (only 47% of patients have a confirmation of the CT findings at angio) [19]. Active contrast extravasation is a sign of active hemorrhage [20]. The use of CT helps in surgical procedure and in AG/AE to be more selective [21, 22]. Contrast blush occurs in about 17% of cases and has been demonstrated to be an important predictor of failure of NOM (more than 60% of patients with blush failed NOM). Its absence on initial CT scan in high-grade splenic injuries does not definitively exclude active bleeding and should not preclude AG/AE [15, 23, 24]. Federle et al. showed that the hemoperitoneum quantification is not related to the risk of NOM failure [20].

#### Non-operative management

*Blunt and penetrating trauma:*

- *Patients with hemodynamic stability and absence of other abdominal organ injuries requiring surgery should undergo an initial attempt of NOM irrespective of injury grade (GoR 2A).*
- *NOM of moderate or severe spleen injuries should be considered only in an environment that provides capability for patient intensive monitoring, AG/AE, an immediately available OR and immediate access to blood and blood product or alternatively in presence of a rapid centralization system and only in patients with stable or stabilized hemodynamic and absence of other internal injuries requiring surgery (GoR 2A).*
- *NOM in splenic injuries is contraindicated in the setting of unresponsive hemodynamic instability or other indicates for laparotomy (peritonitis, hollow organ injuries, bowel evisceration, impalement) (GoR 1A).*
- *In patients being considered for NOM, CT scan with intravenous contrast should be performed to define the anatomic spleen injury and identify associated injuries (GoR 2A).*
- *AG/AE may be considered the first-line intervention in patients with hemodynamic stability and arterial blush on CT scan irrespective from injury grade (GoR 2B).*
- *Strong evidence exists that age above 55-years old, high ISS, and moderate to severe splenic injuries are prognostic factors for NOM failure. These patients require more intensive monitoring and higher index of suspicion (GoR 2B).*
- *Age above 55 years old alone, large hemoperitoneum alone, hypotension before resuscitation, GCS < 12, and low hematocrit level at the admission, associated abdominal injuries, blush at CT scan, anticoagulation drugs, HIV disease, drug addiction, cirrhosis, and need for blood transfusions should be taken into account, but they are not absolute contraindications for NOM (GoR 2B).*
- *In WSES classes II–III spleen injuries with associated severe traumatic brain injury, NOM could be considered only if rescue therapy (OR and/or AG/AE) is rapidly available; otherwise, splenectomy should be performed (GoR 1C).*

#### Blunt trauma

NOM is considered the gold standard for the treatment of patients with blunt splenic trauma (BST) who are hemodynamically stable after an initial resuscitation, in the absence of peritonitis and associated injuries requiring laparotomy [15, 25–28]. In high-volume centers with all facilities, the successful rate of attempted NOM is near 90% [29]. The advantages of NOM over OM were described as lower hospital costs, avoidance of non-therapeutic laparotomies, lower rates of intra-abdominal complications and of blood transfusions, lower mortality and the maintenance of the immunological function, and the prevention of OPSI [27, 30, 31]. Other guidelines have agreed the non-indication of routine laparotomy in hemodinamically stable patients with blunt splenic injury [32, 33].

NOM failure rate is reported to be between 4 and 15% [15, 29, 34–44]. Several risk factors of NOM failure have been reported [15, 29, 34–54].

In several studies, hemodynamic status at the admission has not been considered a significant prognostic indicator for NOM failure and, for this reason, should not be considered an absolute contraindication for NOM [15, 29, 36, 40, 41]. Others reported that the need for red cell transfusions in ED or during the first 24 h [40, 48], hemoglobin and hematocrit levels at admission [40], HIV disease, cirrhosis, and drug addiction [55–57] could affect the outcome after NOM.

The presence of a blush at CT scan has been considered a risk factor for NOM failure only in studies in which AG/AE was not adopted [46, 53]. In addition, the extension of hemoperitoneum at imaging alone cannot be considered an absolute contraindication for NOM [15, 19, 20, 40, 54].

In AAST-OIS injury grades above IV, the failure rate of NOM reaches 54.6% [49], while according to other studies, patients with III–V injury grades could achieve a 87% of success rate [15, 49].

Patients with higher ISS were more likely to fail NOM. According to the literature, two ISS values which were significantly associated with the failure of NOM were above 15 [40] or 25 [37]. This finding is in agreement with the increased risk of associated lesions in higher ISS.

NOM failure in case of missed concomitant abdominal injuries is reported in 1–2.5% of cases [38, 41, 47, 48, 51, 58].

GCS score below 12 alone should not be considered a contraindication for NOM as these patients can be successfully managed non-operatively with a reported overall NOM failure rate near 4.5% [15, 29, 40, 49].

The risk of NOM failure in patients older than 55 years is still debated. A few studies [15, 35, 37, 38, 41, 44, 52, 54] found older age to be a significant prognostic factor for NOM failure [15]. On the other hand, other studies [29, 39, 43, 45, 50] did not find significant differences between patients ≤ 55 and > 55 years. It has been suggested that age> 55 years could be a risk factor for NOM failure only in high AAST-OIS injury grades [36, 38, 49]. Furthermore, the failure of NOM in older patients has been found to be associated with higher mortality rates and longer length of hospital stay than patients < 55 years [44].

Some authors suggested a primary OM in the presence of hypotension in the ED, more than five red blood cell transfused, GCS < 11, high ISS, abdominal AIS > 3, age > 55, and spleen AAST-OIS injury grade > 3. However, it has also been demonstrated that NOM could be successful also in high-risk patients without an increase in complications or mortality rates related to delayed operative interventions [15, 52].

According to larger studies on patients with BST [29], in level I trauma centers, NOM success rate is higher than in level II or III centers. Nevertheless, some authors stated that this might not be associated with the failure of NOM [42, 49].

Finally, severe unstable spleen injuries could ideally benefit from a resuscitation in a hybrid OR with trauma surgeons, in order to increase the spleen salvage rate [59–61].

#### Penetrating trauma

Laparotomy has been the gold standard in penetrating abdominal trauma. Several studies demonstrated as the rate of negative laparotomy ranges between 9 and 14% [62, 63]. For the last 20 years, there has been an increased number of approaches with NOM for gunshot and stab injuries [64, 65].

Carlin et al. in a large series compared penetrating splenic trauma (248 patients) with blunt trauma and found that mortality was not significantly different [66]. However, when the authors compared GSW and SW versus blunt splenic trauma, they found a significant difference in mortality (24 versus 15%, *p* = 0.02). Pancreatic, diaphragmatic, and colic injuries significantly increase the rate of OM approach and mortality for septic complications. The associated pancreatic injuries require frequently spleno-pancreatectomy [66]. Demetriades et al. showed in a prospective study with 225 patients with penetrating splenic injury, the direct relationship between the degree of injury and the possibility of NOM vs. emergency laparotomy [67]. Emergency laparotomy rate was 33% in grade I lesions, and it could increase up to 84% in the grade IV; all splenectomies were in injuries with grade III or higher.

*Indication to angiography and angioembolization:*

- *AG/AE may be performed in hemodynamically stable and rapid responder patients with moderate and severe lesions and in those with vascular injuries at CT scan (contrast blush, pseudo-aneurysms and arterio-venous fistula) (GoR 2A).*
- *In patients with bleeding vascular injuries and in those with intraperitoneal blush, AG/AE should be performed as part of NOM only in centers where AG/AE is rapidly available. In other centers and in case of rapid hemodynamic deterioration, OM should be considered (GoR 2B).*
- *In case of absence of blush during angiography, if blush was previously seen at CT scan, proximal angioembolization could be considered (GoR 2C).*
- *AG/AE should be considered in all hemodynamically stable patients with WSES class III lesions, regardless the presence of CT blush (GoR 1B).*
- *AG/AE could be considered in patients undergone to NOM, hemodynamically stable with sings of persistent hemorrhage regardless the presence of CT blush once excluded extra-splenic source of bleeding (GoR 1C).*
- *Hemodynamically stable patients with WSES class II lesions without blush should not underwent routine AG/AE but may be considered for prophylactic proximal embolization in presence of risk factors for NOM failure (GoR 2B).*
- *In presence of a single vascular abnormality (contrast blush, pseudo-aneurysms and artero-venous fistula) in minor and moderate injuries the currently available literature is inconclusive regarding whether proximal or distal embolization should be used. In presence of multiple splenic vascular abnormalities or in presence of a severe lesion, proximal or combined AG/AE should be used, after confirming the presence of a permissive pancreatic vascular anatomy (GoR 1C).*
- *In performing AG/AE coils should be preferred to temporary agents (GoR 1C).*

The reported success rate of NOM with AG/AE ranges from 86 to 100% with a success rate of AG/AE from 73 to 100% [68–78]. In a large study, Haan et al. suggested that indications to AG/AE were pseudo-aneurysms (PSA) or active bleeding at admission CT scan, significant hemoperitoneum, and high-grade splenic injury [68–70]. More than 80% of grade IV–V splenic injuries were successfully managed non-operatively with AG/AE. A large multicenter study [76] on 10,000 patients found that AG/AE was associated with a reduced odds of splenectomy and that the earlier AG/AE was performed; the less number of patients had splenectomy. A multi-institutional study by Banerjee et al. demonstrated that level I trauma center that had AG/AE rates greater than 10% had significantly higher spleen salvage rates and fewer NOM failure, especially for AAST-OIS grade III–IV injured spleen. AG/AE was also found as an independent predictor of spleen salvage and mortality reduction [78, 79].

A few meta-analyses showed a significant improvement in NOM success following introduction of AG/AE protocols (OR 0.26, 95% CI 0.13–0.53, *p* < 0.002) [54, 80–82]. The failure rate without AG/AE is significantly higher than with AG/AE in AAST-OIS grade IV–V injuries (43.7 vs. 17.3%, *p* = 0.035, and 83.1 vs. 25.0%, *p* = 0.016, respectively) [80].

Specific CT findings can help in the therapeutic decision, and they are correlated with outcomes. As such, patients with PSA and arterovenous fistula showed higher NOM failure rates [21, 22, 53, 83–90].

NOM failure in the presence of contrast blush treated without AG/AE ranges between 67 and 82% [53, 85]. Shanmuganathan et al. reported an 83% accuracy of blush in predicting the need for AG/AE [86]. Marmery et al. showed a 4% of active bleeding vascular injuries in AAST-OIS grade I–II splenic injuries [21, 87]. Intraperitoneal splenic blush exhibited a significantly higher percentage of hemodynamic deterioration during the time required for AG/AE than intra-parenchymal bleedings (*p* < 0.001), suggesting intraperitoneal blush as an independent risk factor for OM [88].

Between 2.3 and 47% CT detected, contrast blush could not be confirmed at the subsequent angiography [89, 90]. The presence of a vascular injury is significantly associated with the splenic injury grade (*p* < 0.0001) [21]. Moreover an analysis on 143 patients with blush at CT scan suggested that an angiographic procedure without embolization increases twofold the risk of re-bleeding and NOM failure [90].

The indication for routine prophylactic AG/AE in high-grade splenic injuries is a matter of controversy [23, 68, 70, 74, 85, 91–93]. Several retrospective and prospective studies recommended the use of AG/AE in all hemodynamically stable patients with high-grade splenic injuries [23, 91–93]. NOM failure rates both with and without prophylactic AG/AE for high-grade injuries are 0–42% vs. 23–67%, respectively, [23, 68, 70, 74, 85, 91].

Controversies exist regarding which kind of lesions should be considered as “high-grade” (AAST III–V or IV–V grade) and should undergo routine AG/AE [23, 68, 91, 92]. It has been reported that NOM could fail in up to 3% of grade III lesions without blush with no AG/AE [23]. Furthermore, no outcome deterioration (in terms of NOM failure, rate of re-bleeding, complications, and mortality) was detected after excluding grade III injuries from routine AG/AE protocol [91]. Therefore, considering the AG/AE-related morbidity of 47% (versus 10% related to NOM without AG/AE) [93] and the fact that widening the selection criteria for AG/AE from grades IV–V to grades III–V may slightly decrease the overall NOM failure rate, patients with grade III lesions without blush should not undergo routine AG/AE.

To date, no randomized comparing proximal and distal embolization are available [94]. In a meta-analysis including 15 retrospective studies, proximal and distal embolization was found to be equivalent with regard to the incidence of major infarctions, infections, and major re-bleeding [95]. However, a significant higher rate of overall minor complications was found after distal AE (2.8–11.6% versus 15.9–25.2%) [95].

Several studies analyzed the morbidity related to AG/AE, to OM, and to NOM without AG/AE [23, 68, 70, 96–103]. The AG/AE major morbidity rates range from 3.7 to 28.5% including re-bleeding, total or subtotal splenic infarction, splenic abscesses, acute renal insufficiency, pseudocysts, and puncture-related complications. The rates for minor morbidities range from 23 to 61%, and they included fever, pleural effusion, coil migration, and partial splenic infarction [70, 96, 102, 103]. All studies [97, 98, 101], but one [93] reported significantly higher complication rates in patients undergone OM (increased rate of death, infectious complications, pleural drainage, acute renal failure, and pancreatitis). In particular, the incidence of infectious complications was significantly higher in the splenectomy group (observation 4.8%, AG/AE 4.2%, splenorrhaphy 10.5%, splenectomy 32.0%, *p* = 0.001) [98].

Some studies analyzed the cost of NOM and AG/AE [104]. They observed that NOM is safe and cost effective, and AG/AE is similar to surgical therapy with regard to cost.

Lastly, AG/AE does not seem to totally compromise the splenic function, and even in presence of an elevated leukocyte and platelet counts, no significant differences in immunoglobulin titers were found between splenic artery AG/AE patients and controls [91]. The spleen due to its intense vascularization could assure the necessary blood to continue its immunological function.

#### Operative management

*Blunt trauma and penetrating:*

- *OM should be performed in patients with hemodynamic instability and/or with associated lesions like peritonitis or bowel evisceration or impalement requiring surgical exploration (GoR 2A)*.
- *OM should be performed in moderate and severe lesions even in stable patients in centers where intensive monitoring cannot be performed and/or when AG/AE is not rapidly available (GoR 2A)*.
- *Splenectomy should be performed when NOM with AG/AE failed and patient remains hemodynamicaly unstable or shows a significant drop in hematocrit levels or continuous transfusion are required (GoR 2A)*.
- *During OM, salvage of at least a part of the spleen is debated and could not be suggested (GoR 2B)*
- *Laparoscopic splenectomy in early trauma scenario in bleeding patients could not be recommended (GoR 2A)*.

Operative management (OM) of splenic injuries should be performed in non-responder hemodynamic instable patients. This condition is frequently observed in high-ISS trauma, in high-grade lesions, and in patients with associated lesions. However, it can be also required in low volume trauma centers or peripheral centers where no intensive care unit or intensive monitoring can be achieve [13, 105, 106]. It has been reported that isolated splenic injury is about 42% of all abdominal trauma [107]. Multiple injuries are reported near 20–30% [107–109]. No sufficient data are available about concomitant vascular and splenic injuries. Associated hollow viscus injuries could be found in 5% of cases; the severity of splenic injury seems to be related to the incidence of hollow viscus injury (1.9, 2.4, 4.9, and 11.6% in minor, moderate, major, and massive injuries, respectively) [110].

The use of splenectomy is decreasing, and the use of splenorrhaphy is rarely adopted (35–24% and 6–1%, respectively) [108, 111]. The attempt to perform a partial splenic salvage is reported in 50–78% of cases, but when NOM fails, splenectomy is the preferred treatment [108, 111].

Laparoscopic splenectomy for trauma is reported only in some cases of hemodynamically stable low-moderate grade splenic injuries [112, 113].

The use of splenic autologous transplantation (i.e., voluntarily leaving pieces of spleen inside the abdomen), to avoid infective risk from splenectomy, has been investigated, but no reduction of morbidity or mortality has been demonstrated [114].

The reported overall hospital mortality of splenectomy in trauma is near 2%, and the incidence of post-operative bleeding after splenectomy, ranges from 1.6 to 3%, but with mortality near to 20% [115].

#### Spleen injuries with concomitant spinal and brain injuries

Particular attention should be posed in managing hemodynamically stable patients with blunt spinal trauma (BST) and severe traumatic brain injury (STBI). A recent study in patients with concomitant spinal and/or brain associated to AAST-OIS grade IV–V spleen injuries reported a general survival benefit of immediate splenectomy over NOM [116]. However, in centers where AG/AE is available (having therefore a lower NOM failure rate of high-grade splenic injuries), immediate splenectomy in patients with severe brain injury does not seem to be associated with an improved survival benefit regardless the grade of injury [116]. It must be highlighted that the differences in definition of hemodynamic instability may represent a bias in this cohort of patients as a few “unstable” patients might have undergone NOM. This data strongly emphasizes the dangers related to poor patient selection for NOM in BST and STBI [34, 49].

*Thrombo-prophylaxis in splenic trauma:*

- *Mechanical prophylaxis is safe and should be considered in all patients without absolute contraindication to its use (GoR 2A).*
- *Spleen trauma without ongoing bleeding is not an absolute contraindication to LMWH-based prophylactic anticoagulation (GoR 2A).*
- *LMWH-based prophylactic anticoagulation should be started as soon as possible from trauma and may be safe in selected patients with blunt splenic injury undergone to NOM (GoR 2B).*
- *In patient with oral anticoagulants the risk-benefit balance of reversal should be individualized (GoR 1C).*

Trauma patients are at high risk of venous thromboembolism (VTE); the transition to a hyper-coagulation state occurs within 48 h from injury [117–119]. Without any prophylaxis, more than 50% may experience deep vein thrombosis (DVT)which substantially increases the risk of pulmonary embolism (PE) whose mortality is about 50% [117, 118]. In trauma patients surviving beyond the first 24 h, PE is the third leading cause of death. Even with chemical prophylaxis, DVT can be detected in 15% of patients. There are currently no standards for the initiation of prophylactic anticoagulation in trauma patients with blunt spleen injuries. A survey-based analysis from ASST reported a growing use of heparin according to the increasing grade of the splenic lesion, and on the contrary, an increasing use of low-molecular-weight heparin (LMWH) in low-grade lesions [120]. Heparin and LMWH can be combined with mechanical prophylaxis; however, mechanical prophylaxis alone in high-grade lesions seems to be preferred by surgeons compared with heparin. Eberle et al. [121] and Alejandro et al. [119] demonstrated no differences between VTE prophylaxis administered within and after 72 and 48 h from trauma respectively, with highest rate of failure in patients with high-grade splenic injury. Bellal et al. [122] found no difference in hemorrhagic complication and NOM failure rate in patients with early (< 48 h), intermediate (48–72 h), and late (> 72 h) VTE prophylaxis. These considerations are referred to selected patients, particularly those without significant head and spinal injuries. As a counterpart, Rostas et al. [117] show that VTE rates were over fourfold greater when LMWH was administered after 72 h from admission.

When trauma occurs in patients under anticoagulants, it is important to consider, if it is necessary, the reversal of their effects in order to avoid thrombotic complication. However, failing to resume anticoagulation in a timely fashion is associated with poor outcomes [123].

Short- and long-term follow-up in NOM (blunt and penetrating)

- *Clinical and laboratory observation associated to bed rest in moderate and severe lesions is the cornerstone in the first 48–72 h follow-up (GoR 1C).*
- *CT scan repetition during the admission should be considered in patients with moderate and severe lesions or in decreasing hematocrit, in the presence of vascular anomalies or underlying splenic pathology or coagulopathy, and in neurologically impaired patients (GoR 2A).*
- *In the presence of underlying splenic pathology or coagulopathy and in neurologically impaired patients CT follow-up is to be considered after the discharge (GoR 2B).*
- *Activity restriction may be suggested for 4–6 weeks in minor injuries and up to 2–4 months in moderate and severe injuries (GoR 2C).*

Splenic complications after blunt splenic trauma range between 0 and 7.5% with a mortality of 7–18% in adults [13]. In children, these incidences are lower [124–127]. The 19% of splenic-delayed ruptures happen within the first 48 h, more frequently between 4 and 10 days after trauma. The risk of splenectomy after discharge ranges between 3 and 146 days after injury, and the rate of readmission for splenectomy was 1.4% [128]. Savage et al. [129] showed that approximately 2% of patients discharged with a non-healed spleen required late intervention. Savage et al. [129] found an average of healing in grades I–II of 12.5 days with a complete healing after 50 days while in grades III–V, 37.2 and 75 days, respectively. In 2–2.5 months, regardless of severity of spleen injury, the 84% of patients presented a complete healing [129]. As a counterpart, Crawford et al. suggested that an early discharge is safe because late failure occurs infrequently [56, 130]. Mortality of late rupture ranges from 5 to 15% compared with 1% mortality in case of acute rupture [40, 131]. In any case, patients undergone NOM should be counseled to not remain alone or in isolated places for the first weeks after the discharge and they should be warned regarding the alert symptoms.

Radiological follow-up is used, but there are not clear information regarding the timing and type of imaging (CT vs. US); thus, imaging follow-up is usually based on clinical judgment and has been widely debated [18, 34, 40, 125, 132–134]. Management strategies that use patient education are more cost effective than to undergo imaging all patients until splenic complete healing.

In the short course (first 24–72 h), observation remains an essential part of low-grade splenic injury (AAST I–II grade); after the admission CT scan, serial abdominal examinations, and hematocrit determination every 6 h are necessary [18]. Clancy et al. [125] showed as PSA were found in patients with grade II, even months after trauma, so they recommended CT scan at 36–72 h in all injuries [129, 131, 132]. Some authors suggest to repeat CT scan only in patients with decreasing hematocrit, in AAST grades III–IV, in patients with subcapsular hematoma, or underlying splenic pathology or coagulopathy, as also in neurologically impaired patients [135].

In the intermediate-long course recent reports recommended that routine post-discharge follow-up abdominal CT is not necessary in low-grade (AAST grade I or II) injuries [132].

More than 50% of patients present a healing at CT scan after 6 weeks, and subsequent image follow-up seems to have no clinical utility [24, 135]. Complete healing of almost all grades is observed 3 months after injury. Lynch et al. [136], in a prospective study, showed that mean time to US healing in AAST grade I, II, Ill, and IV injuries was 3.1, 8.2, 12.1, and 20.7 weeks, respectively. Soffer D. et al. [14] suggest a DUS for splenic lesion follow-up. Some authors have suggested the use of magnetic resonance images [18].

The role of radiological follow-up before returning to normal activity remains controversial. According to some authors, the return to normal activity can occur 3 weeks after splenectomy, and after 2.5–3 months after NOM [126, 134, 136, 137]. Other authors suggested activity restriction of 2 weeks for mild injuries with a return to full activity after 6 weeks, and up to 4–6 months for patients with more severe injuries [120, 129].

## Pediatric patients

### Pediatric splenic trauma

The spleen is the most commonly injured solid organ in pediatric blunt trauma patients (25–30%) [2, 138]. The age limit for pediatric patients is considered for present guidelines to be < 15 years old. While non-operative management of splenic trauma is the mainstay in children, the available clinical guidelines are not universally applied. In urban pediatric hospitals where resources facilitate the non-operative approach, the likelihood of splenic preservation with NOM ranges from 95 to 100% [139].

The Eastern Association for the Surgery of Trauma (EAST) recommends NOM in blunt splenic trauma in all hemodynamically stable children irrespective of the AAST injury grade [140, 141]. The same guidelines recommend a “less is more” approach with respect to imaging studies during admission and follow-up, aiming to reduce the use of CT scan and radiation exposure [140, 142].

NOM seems to be more effective in children, and therefore, it is more commonly used in these patients compared to adults NOM of pediatric splenic trauma which is also associated with reduced cost and lengths of hospital stay, less need for blood transfusions, vaccinations, and antibiotic therapy, as well as higher immunity and reduced rate of infections [142–146].

Even though it is not clear why NOM outcomes are superior in children compared with adults, this phenomenon may be related to certain unique pediatric characteristics (e.g., thicker splenic capsule, higher proportion of myoepithelial cells, more efficient contraction, and retraction of the splenic arterioles [147–152]).

### Clinical presentation in splenic pediatric trauma

The mechanisms of trauma are similar in children and adults. These include motor vehicle and pedestrian injuries as well as sports-related injuries, bicycle injuries, and child abuse [2].

Pediatric injuries differ from adult trauma as the elastic pediatric rib cage may cause a transmission of force into the abdominal compartment [151].

Trauma in neonates represents a rare but unique diagnostic challenge since shock and abdominal rigidity or altered mental status may be the only indications of underlying abdominal injury [2].

In adolescents, the signs of splenic trauma may include the left upper quadrant pain associated with referred left shoulder pain hypovolemic shock or generalized abdominal pain [2].

### Definition of the hemodynamic status in children

According to ATLS, the normal systolic blood pressure in children is 90 mmHg plus twice the child’s age in years (the lower limit is inferior to 70 mmHg plus twice the child’s age in years, or inferior to 50 mmHg in some studies) [5]. Severe blood loss is defined as blood loss greater than 45% of the circulating volume and results in hemodynamic instability. Nevertheless, clinical judgment remains the most important factor in diagnosing an ongoing bleeding [153].

For fluid resuscitation, three boluses of 20 mL/kg of crystalloid replacement should be administered before blood replacement [5, 153]. Massive transfusion protocol in children should be applied with a ratio of 1:1:1 [153]. Transfusion triggers have been debated, and although, there are no class I data to support a specific numerical threshold, it is generally agreed that transfusion should be considered when hemoglobin is less than 7 g/dL [153].

Effective resuscitation is classically indicated by reduction of the heart rate, improved mental status, return of peripheral pulses and normal skin color, increase in blood pressure, and urinary output, as well as increase in extremity warmth [5].

Even though the benefit of tromboelastography (TEG) has not been confirmed in children, recent ATOMAC guidelines suggested that it may be useful in these patients as well (based on adult data) [153].

Diagnostic procedures:

- *The role of E-FAST in the diagnosis of pediatric spleen injury is still unclear (GoR 1A).*
- *A positive E-FAST examination in children should be followed by an urgent CT in stable patients (GoR 1B).*
- *Complete abdominal US may avoid the use of CT in stable patients (GoR 1B).*
- *Contrast-enhanced CT scan is the gold standard in pediatric splenic trauma (GoR 1A).*
- *Doppler US and contrast-enhanced US are useful to evaluate splenic vascularization (GoR 1B).*
- *CT scan is suggested in children at risk for head and thoracic injuries, need for surgery, recurrent bleeding, and if other abdominal injuries are suspected (GoR 1A).*
- *Injury grade on CT scan, free fluid amount, contrast blush, and the presence of pseudo-aneurysm do not predict NOM failure or the need for OM (GoR 1B).*
*Thoracic X-ray* at the admission is recommended in the ATLS guidelines [2, 5].

*Ultrasonography (US)* is the less invasive and is considered the gold standard in trauma, according to the ATLS guidelines especially in Europe [5, 154]. The additional use of DUS or CEUS is helpful and can increase sensitivity for the evaluation of splenic flow and injuries [2]. In patients with low clinical suspicion for splenic trauma, US and CEUS may allow to avoid CT scan [2]. The routine use of CEUS can improve the search of PSA [155].

*FAST (Focused Assessment with Sonography for Trauma)*: The role of FAST for the diagnosis of spleen injury in children is still unclear. Recent Pediatric Emergency Care Applied Research Network (PECARN) data suggest that only 13.7% of pediatric trauma patients with a suspicion of intra-abdominal injuries undergo FAST examination [156]. The sensitivity of this imaging modality in children ranges from 50 to 92%, with a comprehensive meta-analysis suggesting the sensitivity to be around 66% [157–159].

The specificity of this exam is also quite low, and therefore, in a hemodynamically stable patient, a positive FAST examination should be followed by an urgent CT. Bedside FAST may have utility in hemodynamically unstable patients to rapidly identify or rule out intraperitoneal hemorrhage when patients cannot undergo CT.

*Contrast-enhanced computer tomography (CT)* is the gold standard for the evaluation of blunt abdominal trauma [2, 5]. However, patients should be hemodynamically stable, as well as cooperative or sedated. Of note, surgeons should interpret CT findings cautiously before opting for OM because more than 50% of children present with grade III–IV lesions [2, 160]. Taking into account the radiation risk in children, low-dose protocols are preferred (3–6 mSv instead of 11–24 mSv) [2, 5]. APSA guidelines recommend CT scanning in children at risk for injuries that might be missed by FAST, need for surgery, recurrent bleeding, and when other abdominal injuries (such as pancreatic or hollow viscous injury) are suspected [142].

*Non-operative management in splenic injury:*

- *NOM is recommended as first-line treatment for hemodynamically stable pediatric patients with blunt splenic trauma (GoR 2A).*
- *Patients with moderate-severe blunt and all penetrating splenic injuries should be considered for transfer to dedicated pediatric trauma centers after hemodynamic stabilization (GoR2A).*
- *NOM of spleen injuries in children should be considered only in an environment that provides capability for patient continuous monitoring, angiography, trained surgeons, an immediately available OR and immediate access to blood and blood products or alternatively in the presence of a rapid centralization system in those patients amenable to be transferred (GoR 2A).*
- *NOM should be attempted even in the setting of concomitant head trauma; unless the patient is unstable, and this might be due to intra-abdominal bleeding (GoR 2B).*
*Blunt splenic injury:*
- *Blunt splenic injuries with hemodynamic stability and absence of other internal injuries requiring surgery should undergo an initial attempt of NOM irrespective of injury grade (GoR 2A).*
- *In hemodynamically stable children with isolated splenic injury splenectomy should be avoided (GoR 1A).*
- *NOM is contraindicated in the presence of peritonitis, bowel evisceration, impalement, or other indications to laparotomy (GoR 2A).*
- *The presence of contrast blush at CT scan is not an absolute indication for splenectomy or AG/AE in children (GoR 2B).*
- *Intensive care unit admission in isolated splenic injury may be required only for moderate and severe lesions (GoR 2B).*
*Penetrating splenic injury:*
- *No sufficient data validating NOM for penetrating spleen injury in children exist*.

NOM is successful in 95–100% of blunt pediatric trauma patients and has therefore become the gold standard of treatment in children who have sustained an isolated blunt splenic injury and are hemodynamically stable at the time of presentation [139, 161]. AG/AE at present is considered among NOM tools by several authors.

APSA trauma committee recommendations have resulted in reduced ICU stay, hospital LOS, and resource utilization, while achieving superior outcomes [142, 162, 163]. In isolated spleen injuries, ICU stay should be considered in moderate-severe lesions [153, 160].

The CT-based solid organ grading system has not only been used to triage patients but also to administer the most appropriate treatment and to predict outcomes. However, the latter remains controversial [141, 164]. The CT-based solid organ grading system has not only been used to triage patients but also to administer the most appropriate treatment and to predict outcomes. However, the latter remains controversial [154, 161, 165–167]. Therefore, CT scan should not be the only factor guiding the diagnostic process; and some authors use this argument to avoid imaging in a stable patient altogether. Surprisingly, several studies have shown that adherence to APSA guidelines is low in non-pediatric trauma centers [145, 162, 168–172]. Pediatric trauma patients treated in dedicated centers were demonstrated to have higher probability to undergo NOM than those treated in adult trauma centers [145, 162, 168–170]. Mooney et al. and Todd et al. demonstrated that children with splenic injury have a greater chance to undergo splenectomy or laparotomy in general if treated in an adult trauma center [171, 173].

NOM failure rates for pediatric splenic trauma have been shown to range from 2 to 5% [174, 175]. Of note, there is evidence suggesting that the rate of NOM failure peaks at 4 h and then declines over 36 h from admission [174]. Overall, the majority (72.5%) of NOM failures seem to occur during the first week after trauma, with 50% of them happening within the first 3–5 days [37].

Finally, there are no granular data validating NOM for penetrating spleen injury in children. However, reports on successful non-operative management of isolated penetrating spleen injuries in hemodynamically stable pediatric patients do exist [176–178].

*The role of angiography/angioembolization (AG/AE):*

- *The vast majority of pediatric patients do not require AG/AE for CT blush or moderate to severe injuries (GoR 1C).*
- *AG/AE may be considered in patients undergone to NOM, hemodynamically stable with sings of persistent hemorrhage not amenable of NOM, regardless the presence of CT blush once excluded extra-splenic source of bleeding (GoR 1C).*
- *AG/AE may be considered for the treatment of post-traumatic splenic pseudo-aneurysms prior to patient discharge (GoR 2C).*
- *Patients with more than 15 years old should be managed according to adults AG/AE-protocols (GoR 1C).*

The role of AG/AE in the management of pediatric splenic trauma is controversial, and its use varies widely among institutions [164, 179, 180].

Even though AG/AE appears to be a safe intervention, the vast majority of retrospective observational data show that very few pediatric patients with contrast extravasation may benefit from embolization [153, 181].

Therefore, AG/AE may only be considered in carefully selected patients, such as those with high-grade injuries, transient response to resuscitation, and/or persistent blood requirements [182]. Similarly, the role of embolization in the management of pediatric splenic pseudo-aneurysms is also unclear. Of note, PSAs often undergo spontaneous thrombosis and could resolve without any interventions [133, 144, 155, 180, 183]. Some authors proposed a distinction between adolescent of more than 13–15 years old, for which should be applied the adult protocol for AG/AE, and children of less than 13–15 years old that are more vulnerable to OPSI [184, 185]. Moreover, Skattum et al. suggested that if a patient aged less than 15 years old is found to have a PSA on admission CT, contrast-enhanced ultrasound should be performed prior to discharge. If at that time PSA is still present, embolization should be considered [184].

Mortality and major complications are rarely reported following AG/AE [180, 184, 186, 187]. Nevertheless, a post-embolization syndrome (PES), consisting of abdominal pain, nausea, ileus, and fever, seems to occur in 90% of children undergoing AG/AE. This syndrome is usually self-limited and tends to resolve spontaneously in 6 to 9 days [188]. In addition, pleural effusion (9%), pneumonia (9%), and coil migration (4.5%) can also be seen after splenic embolization [184].

Overall, AG/AE seems to preserve splenic function without lasting complications, but most children do not need this intervention [179, 189, 190].

*Operative management in blunt and penetrating injuries:*

- *Patients should undergo to OM in case of hemodynamic instability, failure of conservative treatments, severe coexisting injuries necessitating intervention and peritonitis, bowel evisceration, impalement (GoR 2A).*
- *Splenic preservation (at least partial) should be attempted whenever possible (GoR 2B).*

Indications for laparotomy include hemodynamic instability, ongoing blood loss, or evidence of hollow viscous injury [153, 161, 191–194]. Of note, ATOMAC guidelines recommend surgery if transfusion of 40 mL/kg of all blood products within 24 h (or more than 4 units of blood) fails to stabilize the patient hemodynamically [146, 153]. One percent (1%) of pediatric patients who undergo immediate OM are readmitted for intestinal obstruction within a year [194]. In most cases of OM, splenic partial preservation is possible. Indeed, partial (subtotal) splenectomy or splenorrhaphy are safe and viable alternatives to total splenectomy and can be performed even in high-grade injuries [193, 195–197].

#### Splenic trauma associated with head injuries

Head injury is an important cause of morbidity and mortality in trauma patients of all ages (50–60%). Importantly, head injuries can also result in altered mental status, which can complicate the process of clinical evaluation [198]. Especially in the setting of concurrent head injury, blood pressure and heart rate are poor markers of hemorrhagic shock in pediatric patients [153]. Nevertheless, an analysis of the National Pediatric Trauma Registry suggested that the association of altered mental status from head injury with spleen injuries should not impact the decision for observational management in pediatric patients (< 19 years old) [198].

*Short- and long-term follow-up in splenic trauma (blunt and penetrating):*

- *In hemodynamic stable children without drop in hemoglobin levels for 24 h, bed rest should be suggested (GoR 2B).*
- *The risk of pseudo-aneurysm after splenic trauma is low, and in most of cases, it resolves spontaneously (GoR 2B).*
- *Angioembolization should be taken into consideration when a pesudoaneurysm is found (GoR 2B).*
- *US (DUS, CEUS) follow-up seems reasonable to minimize the risk of life-threatening hemorrhage and associated complications in children (GoR 1B).*
- *After NOM in moderate and severe injuries, the reprise of normal activity could be considered safe after at least 6 weeks (GoR 2B).*

No definitive data exist regarding complication rate and short- and long-term follow-up, and no clear indications regarding the most cost-effective imaging technique (US, DUS, CEUS, CT scan). Initial APSA guidelines [142] recommended bed rest for a number of days equal to the grade of injury plus 1 day [142]. However, recent studies suggest a shorter bed rest of one night in solitary grade I–II splenic trauma and two nights for patients with more severe injuries (grade ≥ III) and stable hemoglobin level [199]. Longer admission should be considered in patients with lower hemoglobin levels on admission, higher injury grade, suspicious of other abdominal injuries (as pancreatic or small bowel injuries), blush on the CT scan, bicycle handlebar injuries, recurrent bleeding, or patients at risk for missed injuries [153, 165].

US or CEUS or DUS follow-up seems reasonable to minimize the risk of life-threatening hemorrhage and its associated complications [200]. General surgeons tend to perform routinely imaging follow-up for children differently from pediatric surgeons that only in 5% of cases suggest imaging follow-up [145, 165, 201].

The APSA guidelines [142] recommended 2–5 months of “light” activity before restart with normal activities and recommended 3 week–3 months of limited activity at home. Some authors suggested the reprise of normal activity even after 4 weeks after III–IV grade injuries. In fact, the risks of delayed splenic rupture and post-traumatic pseudocysts seem to be increase within the first 3 weeks (incidence 0.2 and 0.3%, respectively) [142, 202]. Canadian guidelines suggested a discharge at home after reprise and good toleration of oral intake, able mobilization, and analgesia with oral medications without images before discharge [160]. They reported a 32% of children that did not have any images follow-up without any complications and a restriction of activity no more than 6–8 weeks with a length of activity restriction modulated on the grade of injury [160]. The use of CEUS can improve the diagnosis of PSA that can be found in all grades of injury [155].

Patients and parents psychological involvement after trauma can be related with abdominal pain; for this reason, family and patient education post-discharge should be considered to reduce readmission rate [203].

Infection prophylaxis in asplenic and hyposplenic adult and pediatric patients:

- *Patients should receive immunization against the encapsulated bacteria (Streptococcus pneumoniae, Haemophilus influenzae, and Neisseria meningitidis) (GoR 1A).*
- *Vaccination programs should be started no sooner than 14 days after splenectomy or spleen total vascular exclusion (GoR 2C).*
- *In patients discharged before 15 days after splenectomy or angioembolization, where the risk to miss vaccination is deemed high, the best choice is to vaccinate before discharge (GoR 1B).*
- *Annual immunization against seasonal flu is recommended for all patients over 6 months of age (GoR 1C).*
- *Malaria prophylaxis is strongly recommended for travelers (GoR 2C).*
- *Antibiotic therapy should be strongly considered in the event of any sudden onset of unexplained fever, malaise, chills, or other constitutional symptoms, especially when medical review is not readily accessible (GoR 2A).*
- *Primary care providers should be aware of the splenectomy/angioembolization (GoR 2C).*

OPSI are defined as fulminant sepsis, meningitis, or pneumonia triggered mainly by *Streptococcus pneumoniae* (50% of cases) [204, 205] followed by *H. influenzae* type B and *N. meningitidis*. OPSI is a medical emergency. The risks of OPSI and associated death are highest in the first year after splenectomy, at least among young children, but remain elevated for more than 10 years and probably for life. The incidence of OPSI is 0.5–2%; the mortality rate is from 30 to 70%, and most death occurs within the first 24 h. Only prompt diagnosis and immediate treatment can reduce mortality [2, 204, 206, 207]. Asplenic/hyposplenic children younger than 5 years old have a greater overall risk of OPSI with an increased death compared with adults [204, 208]. The risk is more than 30% in neonates [2]. Evidence exist regarding the possible maintaining of the function by the embolized spleen (hyposplenic patients) however is reasonable to consider it as less effective and proceed with vaccination as well [179, 189, 190].

Vaccination against flu is recommended annually for asplenic/hyposplenic patients over 6 months of age. Prevention of influenza may decrease the risk of secondary bacterial infection, including pneumococcal infection [207, 208].

Ideally, the vaccinations against *S. pneumoniae*, *H. influenzae* type B, and *N. meningitidis* should be given at least 2 weeks before splenectomy [2]. Patients should be informed that immunization can only reduce the incidence of OPSI (vaccines so far available do not allow an exhaustive coverage neither for *S. pneumoniae*—23 of 90 serotypes are included—nor for *N. meningitidis*—5 of 6 serotypes) (Table 5).

**Table 5:**
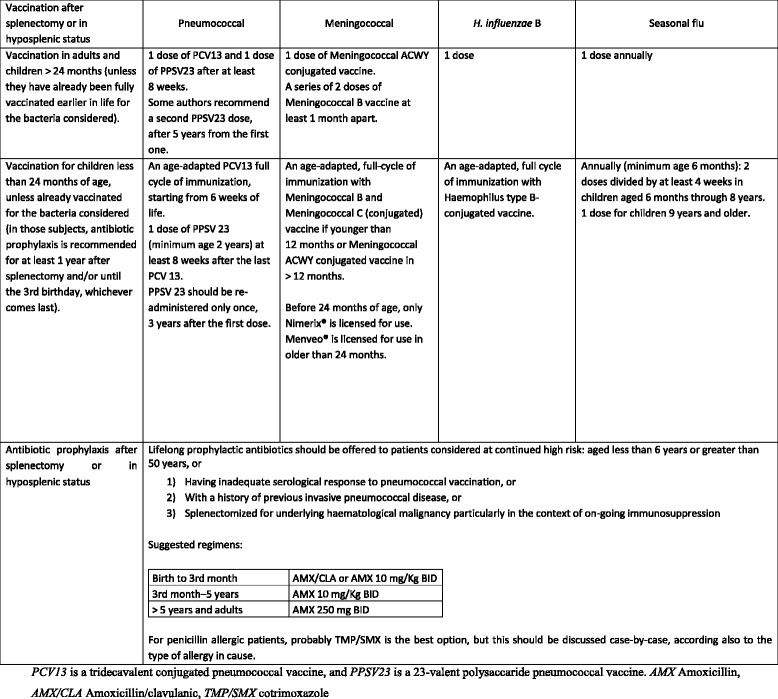
Vaccinations and antibiotic prophylaxis after splenectomy or hyposplenic status

In traumatic patients, the correct time for vaccination should be not less than 14 days after splenectomy; in fact, before 14 days, the antibody response is supposed to be suboptimal [204, 206, 209]; after that interval, the earlier the better. In asplenic/hyposplenic patients discharged before 15 days, where the risk to miss the vaccination is deemed high, the first vaccines should be given before discharge [206, 210]. The Centre for Disease Control in 2016 proposed the last updated recommendations [211]. Most episodes of severe infections occur within the first 2 years after splenectomy, and for this reason, some authors recommend at least 2 years of prophylactic antibiotics after splenectomy. However, the duration of antibiotic prophylaxis is controversial.

Community physicians should be aware of the asplenic/hyposplenic condition, in order to provide them with the most appropriate level of care.

Asplenic/hyposplenic patients should be given an antibiotic supply in the event of any sudden onset of unexplained fever, malaise, chills, or other constitutional symptoms, especially when medical review is not readily accessible. The recommended options for emergency standby in adults include the following: (a) Amoxycillin, 3 g starting dose followed by 1 g, every 8 h; (b) Levofloxacin 500 mg every 24 h or Moxifloxacin 400 mg every 24 h (for beta-lactam allergic patients).

The recommended emergency standby treatment in children is Amoxycillin 50 mg/Kg in three divided daily doses. For beta-lactam allergic patients, an alternative should be proposed by a specialist (fluoroquinolones are generally contraindicated in children, but due to the possible severity of OPSI, they might still be considered).

Antibiotic prophylaxis is necessary in patients with asplenia/hyposplenia who are bitten by dogs and other animals because of increased risk of severe sepsis (Amoxycillin/Clavulanic acid for 5 days) [205, 207, 208].

If the patient is being treated in an outpatient setting, he/she should be referred immediately to the nearest emergency department. Clinical deterioration can be rapid even after antibiotic administration. Antibiotics should be modified once blood culture results become available [208]. Failures of antibiotic prophylaxis have been reported, so patients should be warned that prophylaxis reduces but does not abolish the risk of sepsis.

Due to the increased risk of severe malaria, asplenic/hyposplenic travelers to endemic areas should receive an adequate pre-departure counseling, regarding both measures aimed at reducing the exposure to mosquitos’ bites and chemoprophylaxis.

## Conclusions

The management of spleen trauma must be multidisciplinary and must keep into consideration the physiological and anatomical derangement together with the immunological effects. Critical and operative decisions can be taken more effectively if both anatomy of injury and its physiological effects, and the associated lesions are considered especially considering the modern tools for integrated bleeding management. The treatment algorithm must differ within adults, and children these lasts should always be treated in dedicated trauma centers.

## Abbreviations

AAST: American Association for Surgery for Trauma
AG/AE: Angiography/angioembolization
AIS: Abbreviated injury score
AMX: Amoxicillin
AMX/CLA: Amoxicillin/clavulanic
APSA: American Pediatric Surgical Association
ATLS: Advanced Trauma Life Support
BE: Base excess
BST: Blunt spinal trauma
CEUS: Contrast-enhanced US
CT: Computerized tomography
DPL: Diagnostic peritoneal lavage
DUS: Doppler US
DVT: Deep venous trombosis
EAST: Eastern Association for the Surgery of Trauma
ED: Emergency Department
E-FAST: Extended focused assessment sonography for trauma
GCS: Glasgow Coma Scale
ICU: Intensive Care Unit
ISS: Injury severity score
LE: Level of evidence
LMWH: Low-molecular-weight heparin
LOS: Length of hospital stay
MTP: Massive transfusion protocols
NOM: Non-operative management
OIS: Organ Injury Scale
OM: Operative management
OPSI: Overwhelming post-splenectomy infections
OR: Operating room
PE: Pulmonary embolism
PES: Post-embolization syndrome
PSA: Pseudoaneurism
TBI: Traumatic brain injury
TEG: Thrombo-elastography
TMP/SMX: Cotrimoxazole
US: Ultrasonography
VTE: Venous thromboembolism
WSES: World Society of Emergency Surgery
